# Professional nurses’ experience in providing nursing care to women experiencing gender-based violence: A caring presence study

**DOI:** 10.4102/hsag.v26i0.1658

**Published:** 2021-09-28

**Authors:** Avhatakali Mphephu, Emmerentia du Plessis

**Affiliations:** 1 Department of Health, Tshilidzini Hospital, Thohoyandou, South Africa; 2 NuMIQ Research Focus Area, Faculty of Health Science, North-West University, Potchefstroom, South Africa

**Keywords:** caring, caring presence, experience, gender-based violence, professional nurses, rural area, women

## Abstract

**Background:**

Women in a sub-district of one of the most rural provinces in South Africa are at a high risk of experiencing gender-based violence. Professional nurses are at the frontline of providing healthcare to these women. Caring presence is a valuable resource to professional nurses in these settings.

**Aim:**

To explore and describe the experiences of professional nurses in providing nursing care to women experiencing gender-based violence.

**Setting:**

A primary health clinic, community health centre, out-patient department and emergency department in the rural sub-district.

**Methods:**

An interpretive phenomenological design was applied. Purposive sampling was used and semi-structured one-on-one interviews were held with professional nurses. Interpretive phenomenological analysis was done and data saturation was achieved (*n* = 15).

**Results:**

Participants were willing to provide nursing care but worked in difficult environments, and their level of competence influenced how they engaged with these women. They realised that the lifeworld of the women made it difficult for them to disclose that they are experiencing gender-based violence. Participants emphasised the importance of multidisciplinary and multi-sectoral collaboration. A final theme, caring presence, also emerged.

**Conclusion:**

Participants felt compassion and were willing to provide nursing care. However, they experienced reluctance due to hindrances that limited them in connecting with and attuning to the women. This left them feeling frustrated, and with a deepened sense of empathy, as they realised how deeply the women are suffering. Recommendations were formulated.

**Contribution:**

This study revealed nurses’ need to be guided in providing relational care to women who are experiencing gender-based violence. Based on the findings, it is recommended that infrastructure should be updated to ensure private and safe spaces for women, debriefing and training should be provided and multidisciplinary collaboration should be strengthened. Policy for improved referral systems, the assessment and management of women experiencing gender-based violence and the wellness of professional nurses should be developed.

## Introduction

Women in rural South Africa are at higher risk for gender-based violence (South Africa 2019). The Vhembe region in Limpopo continues to report the highest number of women experiencing gender-based violence (Ibid. 2018). Women in this region are exposed to sexual, physical, psychological and emotional violence, and economical abuse (Alshammari, McGarry & Higginbottom 2018). Extreme cases, such as homicide related to gender-based violence, are reported (Mukwevho 2017). There is a global call for a multi-sectoral plan of action to address gender-based violence (Joyner 2016:961). This is critically important, as the consequences of gender-based violence not only include physical trauma, but also emotional and psychological trauma such as post-traumatic stress disorder (PTSD), major depression, anxiety disorders, suicide and increased substance use (Santos et al. 2018).

At the heart of the call to address gender-based violence are professional nurses who have frontline contact with women experiencing gender-based violence at primary healthcare clinics (PHCs), community health centres (CHCs), the outpatient department (OPD) and the emergency department (ED). Professional nurses play a significant role in identifying gender-based violence and in providing nursing care. Professional nurses are limited in this role by a lack of time and privacy, the fear of accidentally offending the women and a lack of self-confidence (Clark, Bradbury-Jones & Taylor 2017). Guruge (2012) also mentioned barriers namely: a high workload, lack of knowledge and skills, fear for personal safety, language, lack of communication skills, the relatively low status of nurses in the health system hierarchy and lack of support.

The practice of caring presence may offer a valuable solution. A caring presence is acquired when nurses help patients holistically and are open to the patient. It includes self-actualisation and co-creating a conducive environment, and it is associated with practices such as listening and touch (Mohammadipour et al. 2017). Caring presence requires of nurses to attune to and connect with patients in a professional nurse-patient relationship, leading to relational care (McMahon & Christopher 2011). Relational care is an attribute of caring presence and occurs when the nurse reaches out to the patient, and provides nursing care through and within a professional relationship in which the needs of the patient is attended to (McMahon & Christopher 2011). If the acquisition of a caring presence is taught to professional nurses as the first-contact healthcare providers to women experiencing gender-based violence, it can bring positive outcomes. It can lead to a decrease in pressure on the women so that they feel accepted, relieved and able to talk about gender-based violence issues and a decrease in consequent stress-related mental illness (Mohammadipour et al. 2017).

### Problem statement

An alarming increase in gender-based violence is noted, especially against women living in rural areas (Clark et al. 2017), such as the Vhembe district in Limpopo. Most of these women are not attended to comprehensively in OPDs, despite the mission to ensure quality, equity and accessible patient care (Department of Health 2017). This situation leads to women not disclosing that they are experiencing gender-based violence, leading to an increase in complications like stress-related mental illnesses (Alshammari et al. 2018; Clark et al. 2017).

Professional nurses working in frontline units, such as PHCs and CHCs, OPDs and the ED, can be a core source of assistance to women experiencing gender-based violence (Baird et al. 2018). However, it seems that nurses feel unprepared and are reluctant to engage in conversations on gender-based violence with women (Ibid. 2018). The first author’s experience confirms this trend. Working as a professional nurse at the ED of a public hospital in the Vhembe district, she observed that women experiencing gender-based violence are not offered quality time, even to the extreme where they are being ignored and mocked, and only their physical injuries are attended to.

On the contrary, the caring presence approach advocates for relational care and requires that the nurse displays readiness to connect with and attune to the patient (McMahon & Christopher 2011). Taking the above into consideration, it made sense that the experiences of professional nurses in providing nursing care to women who experience gender-based violence should be explored. It was argued that this would give insight into both their inner wisdom and their difficulties in relating to women experiencing gender-based violence so that recommendations could be formulated on how professional nurses working in PHCs, CHCs, OPDs and EDs can be guided to provide more relational care to women experiencing gender-based violence in a rural area such as the Vhembe district.

These arguments lead to the following research question and sub-questions:

What are the experiences of professional nurses working at PHCs, CHCs, OPDs and EDs in the rural sub-district, of providing nursing care to women experiencing gender-based violence?

- What do professional nurses experience while providing nursing care to these women?
- What is the readiness of professional nurses to attune to and connect with these women?
- How can professional nurses be guided to provide relational care to these women?

## Research aim and objectives

This study aimed to explore and describe the experiences of professional nurses working in PHCs, CHCs, OPD and the ED in the rural sub-district of providing nursing care to women experiencing gender-based violence.

The objectives were to:

- Explore and describe the experiences of professional nurses in providing nursing care to these women.
- Explore and describe the readiness of professional nurses to connect with and attune to these women.
- Explore and describe how professional nurses can be guided to provide relational care to these women.

## Research methodology

### Design

A qualitative, interpretive phenomenological design was applied, as a human experience was explored and described to expand nursing knowledge (Smith & Osborn 2007:54). This design seeks to gain a deeper understanding from the perspective of the participants, unveiling deeper meanings in the context of the mentioned lived experience (De Vos et al. 2019:308). The findings of the research were interpreted keeping the context of the study in mind; the findings were immersed in the concept of ‘caring presence’ and the first author’s knowledge of the context was acknowledged as an additional insight during data analysis.

### Study context

The study was conducted in Limpopo. This province consists of five districts, with the Vhembe district reporting the highest rate of gender-based violence (South Africa 2015). The Vhembe district has four health sub-districts, and is rated as one of the poorer districts of the province. The sub-district relevant in this study is rated the most populated area in the relevant province, with one specialised psychiatric hospital, one regional hospital, one district hospital, three CHCs, 39 PHCs and five mobile clinics, with an extremely high number of PHC visits (South Africa 2015). Community members often have to walk far to reach healthcare clinics, and transport is complicated by unreliable public transport and unworthy roads.

### Sampling process

One district hospital was included in the study (with one OPD and one ED), one CHC with the highest number of patients and one PHC with the highest number of patients in the rural sub-district. Professional nurses working at these institutions, with at least two years of working experience in these institutions, would have experienced providing nursing care to women experiencing gender-based violence. The population thus comprised professional nurses working in a PHC, CHC, OPD or ED in the rural sub-district.

Participants were selected through purposive sampling. Inclusion criteria involved that both male and female professional nurses were included and they were working in a PHC, CHC, OPD or ED in the rural sub-district. Only professional nurses with at least two years of working experience in the Vhembe district and who were providing nursing care to women experiencing gender-based violence were selected to participate. Exclusion criteria were nurses of other categories and nurses working in the Thohoyandou area of the Thulamela sub-district. This is the area where the first author works as a professional nurse.

The sample size was determined by data saturation (Brink et al. 2018:128), which was reached at 15 participants. Data saturation became evident when no new information emerged from the interviews and was confirmed during data analysis. Fourteen (14) female professional nurses and one male professional nurse participated. They were between the ages of 24 and 55 years, and their work experience in the Vhembe district ranged between 2 and 20 years. Eight of the participants had a nursing diploma, whilst seven had a nursing degree. Two participants had additional specialisation in trauma nursing, three in primary health care, and one in midwifery and primary healthcare.

### Data collection

Semi-structured, one-on-one interviews were conducted by the first author. An interview schedule was developed based on the research questions, research objectives, relevant literature on caring presence, and knowledge of the context:

- What is it like to provide nursing care to women experiencing domestic violence?
- What makes it easy for you to attune to and connect with women experiencing domestic violence?
- What makes it difficult for you to attune to and connect with women experiencing domestic violence?
- How would you like to be guided to provide care through relationship to women experiencing domestic violence?

The first author gained experience in research interviewing by completing a module in research methodology and by conducting role-play interviews. The first actual interview was seen as a trial run interview and was submitted to the second author for quality control.

The interviews were conducted in a safe and comfortable private room and were audio-recorded that lasted between 30 and 45 min (De Vos et al. 2019:351). Strict COVID-19 prevention precautions were taken. Field notes, including descriptive, reflective and demographic notes, were recorded directly after the interviews and read in conjunction with the data during data analysis. The one-on-one semi-structured interviews were conducted over one-and-a-half months during 2020.

### Data analysis

The audio-recorded interviews were transcribed *verbatim*. Data analysis entailed an initial identification (becoming aware) of significant words or phrases, followed by an interpretive phenomenological analysis as described by Smith and Osborne (2007:66–75). The researchers carefully read through each transcript and reflected on the deeper meaning of the participants’ responses in terms of what they shared, their context and the integral concept of ‘caring presence’ (Smith & Osborne 2007:40). They identified the essence of phrases which were grouped to form emergent themes. An independent co-coder was involved. The researchers and the co-coder had discussions to reflect on the themes and sub-themes, and to reach a consensus on the findings.

### Trustworthiness

The researcher ensured the trustworthiness of the research by using criteria for establishing trustworthiness, that is, truth value, applicability, consistency and neutrality (Lincoln & Guba 1985:290).

To ensure truth value and credibility, the strategies of peer examination and prolonged engagement were applied. Through prolonged engagement, the first author became aware of how personal and sensitive the topic of the study was for the participants. The participants became emotional whilst sharing their experiences, and in some instances, they disclosed that they had also experienced gender-based violence. Four participants were referred for further counselling. The first author paused the interviews as needed. In cases where the participant decided to continue with the interview, another date and time was arranged for the interview. The researchers thus acknowledge that the findings are deep personal accounts shared by the participants, which is in line with the interpretive phenomenological approach of this research.

Furthermore, the criteria of applicability, by ensuring transferability, were met by providing a thick, dense description of the research and by using purposive sampling. Consistency and dependability were achieved through a dense description, taking rich field notes and involving an independent co-coder. These strategies also contributed to ensuring neutrality and conformability.

### Ethical considerations

Ethical approval was granted by the Health Research Ethics Committee (HREC: reference 00444-19-A1) of the North West University. Permission to conduct the research was obtained from the Department of Health Limpopo and district and sub-district healthcare managers of the Vhembe district, Thulamela sub-district, and the chief executive officer of the selected hospital. After obtaining ethical approval and the necessary permission from authorities, the first author contacted the nursing managers of the PHC, CHC and the OPD and ED to obtain their goodwill permission. At each of these units, the nursing manager was requested to identify a mediator and an independent person, for example, a social worker or an administrative officer who is not in a power relationship with the potential participants. The mediator was requested to assist with the recruitment of participants. All the key role players involved in the study signed a confidentiality agreement. Written, voluntary informed consent was obtained by the independent person.

### Limitations

Despite the rich information given by the participants, the fact that the participants agreed to mostly use English in the interviews led to some information possibly not being revealed because of limited English language ability. Furthermore, the findings are limited to one district in Limpopo, to EDs, OPDs, CHCs and PHCs and professional nurses. Also, the findings are limited to the viewpoint of professional nurses, excluding the experience of patients and other stakeholders.

## Findings and discussion

The section describes the experiences of the participants of providing nursing care to women experiencing gender-based violence. Furthermore, the themes and sub-themes are discussed, supported by direct quotations from the participants. Each theme is embedded in the existing literature.

Table 1 indicates the five themes and related sub-themes that emerged from the data.

**TABLE 1:** Participants’ experience in providing nursing care to women experiencing gender-based violence.

Themes	Sub-themes
Participants are willing to provide quality patient care, but work in a difficult environment	Infrastructure
	Shortage of nursing staff
Participants experienced that their level of competence, their attitude, work overload and communication skills influenced how they engage with women experiencing gender-based violence	Lack of relevant skills
	Attitude
	Work overload
	Communication skills
Participants realised that the lifeworld of women experiencing gender-based violence made it difficult for them to disclose gender-based violence	Culture
	Fear of in-laws
	Poverty and insecurity
Collaboration with the multidisciplinary team and other stakeholders	Multidisciplinary team
	Other stakeholders
Caring presence	Willingness to attune to and connect with women experiencing gender-based violence
	Spending quality time with women experiencing gender-based violence
	Willingness to be personally present
	Willingness to provide holistic care

*Source:* Mphephu, A., 2021, ‘Professional nurses’ experience when caring for women who are experiencing intimate partner violence: A caring presence study’, Masters - Mini-dissertation, North-West University, Potchefstroom.

### Theme 1: Participants are willing to provide quality patient care but work in a difficult environment

#### Infrastructure

The infrastructure of workplaces was experienced as not conducive to taking full assessments, especially for sensitive issues like gender-based violence. There were no separate rooms available for interview women experiencing gender-based violence. These circumstances made it difficult for the participants to comprehensively assess the women and have in-depth discussions as they were aware that they could offer only limited privacy and confidentiality:

‘The other issue is the space. You find out that the cubic you are using while speaking to this patient, the next patient could hear. So it makes it difficult for me to connect with this other patient because they will be having fears of being judged, what if I say this, they will say I am being abused.’ (Participant 8, female, 26 years old, 3 years’ experience, nursing degree, CHC)

#### Shortage of nursing staff

Participants experienced that a shortage of nursing staff hindered them to care for women experiencing gender-based violence. They were understaffed and there were no nurses specifically trained to assess for gender-based violence. Staff shortages lengthened the waiting time of the women experiencing gender-based violence.

‘Because we have shortage of staff you will find out that other patients are waiting for you outside. So for that reason you cannot spend a lot of time with one patient and get details about what happened to them.’ (Participant 1, female, 30 years old, 7 years’ experience, ED)

Similar to these findings, Colombini, Dockerty and Mayhew (2017) reported that the infrastructure in hospitals and PHC settings is often of poor quality with no privacy, leading women to feel uncomfortable disclosing gender-based violence. Authors such as Alshammari et al. (2018:237–253) and Mukwevho (2019) also listed the shortage of nurses in both PHCs and the hospital as one of the barriers to high-quality nursing care to women experiencing gender-based violence.

### Theme 2: Participants experienced that their level of competence, their attitude, work overload and communication skills influenced how they engage with women experiencing gender-based violence

#### Lack of relevant skills

Participants revealed that they are trying to use their skills but they feel that they are not competent enough when it comes to the assessment and management of women experiencing gender-based violence. They feel reluctant and afraid to approach the women when they suspect gender-based violence. They needed training on relevant knowledge and skills. Some of them who experienced or witnessed gender-based violence themselves advised the women based on their experience, but they still feel that they need formal training on how to support these women.

‘We are just using our own experience to say this has worked for me some years back let me try to apply it now. If we get workshops on assisting troubled women in relationships or at home, who might be abused or going through domestic violence, we can assist them properly. It can also become quick for them to get assistance. I need formal and proper skills to assist.’ (Participant 7, male, 45 years old, 5 years’ experience, CHC)

#### Attitude

Despite their feelings of incompetence, participants tried to display positive attitudes to win the trust of the women and enable them to disclose their status of experiencing gender-based violence. They tried to avoid being judgemental when helping women experiencing gender-based violence. They indicated that women will not be open if professional nurses display a negative attitude, but that they speak freely when they are welcomed warmly with a positive, friendly smile.

‘I make sure I put my all when attending to these women because I know it is not safe out there not only in the community but even in the comfort of our own home, because they are being violated every day. So I make sure that when the woman comes in being beaten up, I connect with them all the way. I don’t judge them, I always try to be neutral and when they are crying I try to comfort them.’ (Participant 8, female, 26 years old, 3 years’ experience, nursing degree, CHC)

#### Work overload

Participants revealed that work overload affected their interest and willingness to provide quality patient care to women experiencing gender-based violence. They needed quality time to build trust and create a safe space for women to open up. Participants emphasised that work overload caused them to miss the real problem of the women.

‘There are so many patients we see in a day; you end up not probing enough because you can see the queue is long and there are lots of patients to attend to. So you end up not seeing those signs of abuse because they do not say that they are being abused. You just pick it up when you are probing that this woman is being abused.’ (Participant 10, female, 35 years old, 8 years’ experience, nursing degree with midwifery and PHC specialisation, CHC)

#### Communication skills

Although they were trying to implement the communication skills that they acquired through experience, participants were still not comfortable with the way they communicate with women experiencing gender-based violence. This was especially their experience when the women are not opening up and they have to probe. They feel the need to be trained on the correct way to communicate with women experiencing gender-based violence.

‘If you are having this good communication skills in assessing skills, you can go deep into everything and you may find that, you know, this person is having this type of problem. It’s not that deep inside is just that her problem is not solved.’ (Participant 15, female, 48 years old, more than 10 years’ experience, nursing diploma, OPD)

Alshammari et al. (2018:237–253) confirm these finding by emphasising the importance of continuous training on gender-based violence and relevant communication skills. Sundborg et al. (2012) also found that the attitude of healthcare providers influences how a woman will be assessed and managed. Colombini et al. (2017) confirm that effective communication skills promote disclosure.

### Theme 3: Participants realised that the lifeworld of women experiencing gender-based violence made it difficult for them to disclose

#### Culture

Participants experienced that, in their culture, women are considered as the property of their husband and their in-laws. It is as if women cannot think, cannot plan – a man and his sisters (*vho makhadzi*) are always right, have authority and can make decisions. Women are socialised to be submissive and not to tell other people what is going on within the family. Participants also revealed that it is culturally accepted when a man assaults his wife, but it is taboo for a woman to beat her husband; she may even lose her marriage. They also indicated that when a woman is getting married, a bridal shower is conducted wherein the woman is given rules and norms such as: in this house, no one came back after marriage (*mbuyavhuhadzi);* the grave of the wife is at her marriage place; when things go wrong in your family, you have to calm them *(vhuhadzi ndi nama ya thole, ya fhufhuma ndi a fhunzhela)*. All these rules normalise abuse in the family and women do not report it.

‘According to our culture, we are not supposed to voice out when the husband is beating you. According to our culture, it is normal when the man is beating you and that it makes the woman not to verbalise. They are not free to verbalise because they think maybe it is normal, they are not disclosing.’ (Participant 13, female, 47 years old, 15 years’ experience, nursing degree, OPD)

#### Fear of in-laws

Participants revealed that women are afraid of their in-laws (the mothers-in-law and the sisters-in-law). They tend to tell lies because they are afraid that if their husband is arrested, then his relatives will turn their back against them.

‘Women are told not to say things about their marriages. They are afraid to lose their husband, they are afraid to be single. It makes it difficult to attune to these women experiencing domestic violence, also because of our social economic status the poverty, these women think that if I open up and the man is arrested who will support us.’ (Participant 10, female, 35 years old, 8 years’ experience, nursing degree with midwifery and PHC specialisation, CHC)

#### Poverty and insecurity

Participants mentioned that a large number of women from their areas are poorly educated; they are unemployed and are considered as housekeepers who should serve their in-laws and husband to be provided with food, shelter and clothing. They added that the opinions of women in the rural areas are not considered during decision-making, and they have to endure hardship. Participants experienced compassion towards these women and felt driven to empower them.

‘Like I said before those women don’t speak up and the main reason for them not to speak up is that most of them are not working, they depend on their husband.’ (Participant 6, female, 40 years old, 5 years’ experience, nursing diploma with PHC specialisation, PHC)

In the same vein, Vranda et al. (2018) indicated that African women reported that they are being abused and insulted in front of their in-laws by their husbands whilst their in-laws reported their mistakes to their husbands. Field et al. (2018) also confirm that women in rural areas are insecure economically, educationally and in food supply, and thus are vulnerable to gender-based violence – and the act of abuse by her husband is socially acceptable and normalised.

### Theme 4: Collaboration with the multidisciplinary team and other stakeholders

#### Collaboration with the multidisciplinary team

Participants were able to refer and present women experiencing gender-based violence to other multidisciplinary team members, but they still feel that it is not enough. Sometimes, they have to stay with a referred woman for hours before being attended to by the concerned team members. Furthermore, these women depend on their husbands financially who would not provide the finances for them to travel long distances to the hospital where the relevant multidisciplinary team members are situated. Participants thus identified the need for closer collaboration between multidisciplinary team members and a more accessible referral system for women experiencing gender-based violence.

‘We must try to invest in multidisciplinary teams and should be involved so that we know how to handle the domestic violence patients better refer what needs to be done.’ (Participant 10, female, 35 years old, 8 years’ experience, nursing degree with midwifery and PHC specialisation, CHC)

#### Collaboration between different stakeholders

Participants experienced that there is a need for interaction amongst different stakeholders that should provide comprehensive care to women experiencing gender-based violence as this will lead to quality holistic care. They expressed the need to work collaboratively with the police department, the justice department, the social work department, the department of education and the media.

‘If health centres knows there is someone experiencing domestic violence, they know exactly who to call so that we can fast-track this process of getting these women to go to trauma centre and wait for 24 hours to wait for somebody who is coming from Thohoyandou, so if we get that department links it can be so fast, health care centres and hospital know where to refer and who to talk to if something is not happening.’ (Participant 7, male, 45 years, 5 years’ experience, nursing degree, CHC)

‘At the schools’ level I think if we can have curriculum of domestic violence starting from primary level, secondary level even in tertiary. I think that this can be helpful because as a child will grow up knowing that these things of domestic violence it’s not good I think that is the best way that we can be able to overcome this issue of domestic violence.’ (Participant 3, female, 27 years, 5 years’ experience, nursing degree, ED)

‘I think it can help because when explaining it in the radio there are many women who are being abused by their husband and they’re afraid to talk maybe they can feel free to come.’ (Participant 5, female, 24 years old, 2 years’ experience, nursing diploma, PHC)

Guruge (2012:6) also found that there is a lack of proper communication amongst nurses and other multidisciplinary team and this deprives women experiencing gender-based violence of quality healthcare. Hart and Klein (2013:93) also supported the findings by explaining the importance of educating people about gender-based violence through the media.

### Theme 5: Caring presence

#### Willingness to attune to and connect with women experiencing gender-based violence

Many participants verbalised their willingness to attune to and connect with women experiencing gender-based violence, though they acknowledged that workload, poor infrastructure and lack of skills hindered them to do so. When they were able to attune to the needs of the women and connect with them, they experience the women opening up to them and disclosing that they are experiencing gender-based violence.

‘It is painful to me but I have to be strong for the woman to see that she is getting full assistance from the person who is really willing to assist her but deep down its painful, but I do all my best for her to be comfortable and feel free.’ (Participant 2, 33 years old, 6 years’ experience, nursing diploma with trauma specialisation, ED)

#### Spending quality time with women experiencing gender-based violence

Participants were aware that women experiencing gender-based violence need enough time to speak out. They are willing and try to spend their time with the women experiencing gender-based violence, for example, when taking their history. They revealed that it is not easy to determine the actual problem from the women experiencing gender-based violence; time is needed to probe for underlying information and to comfort them.

‘I just open up and make time to be with them and try to listen what is really happening in their life, if I am not busy, if we have enough staff in the ward, I make sure I put my all when attending to these women. The issue is lack of time and shortages because in our hospital we have the issue of shortage of doctors, we spend less time with them, trying to push the queue outside, you find out that when you are about to move to the next patient, this one start to open up, and you tell them you had your chance but you didn’t want to talk and is not because we don’t care but is because of the shortage of staff.’ (Participant 8, female, 26 years old, 3 years’ experience, nursing degree, CHC)

#### Willingness to be personally present

Participants indicated that they are willing to be personally present for the women experiencing gender-based violence, but they also indicated that the issues of shortage of staff and work overload prevent them from being personally present for the women experiencing gender-based violence, as discussed under themes 1, 2, 3 and 4. The researcher managed to identify the urge to be personally present for the women experiencing IPV during the interviews through non-verbal communication.

‘Then the other thing is that I just want to be free to them and then as a nurse, I don’t want people to see me just as a nurse they have to see me as a caring person as sister and as a mother that’s the thing that makes it easy for me to connect with those people the way I socialise with them I want them to be free I don’t want them to see my profession and makes it difficult for them to connect with me and to be open to me when I communicate with them I make sure that, no this is my sister this is my mother I communicate in a way that the person will feel free and say I’ve got a sister I’ve got a mother who is taking care of me.’ (Participant 3, female, 27 years, 5 years’ experience, nursing degree, ED)

#### Willingness to provide holistic care

Participants show that they are willing to provide holistic care to women experiencing gender-based violence although some of the issues discussed above challenge them. They suggested interaction with other multidisciplinary teams and other departments to provide holistic care and indicated the need for proper training for them to provide holistic care:

‘I just open up and make time to be with them and try to listen to them, give them that smile to feel appreciated and it means I have to hold their hand during the assessment. I think as nurses we need more of in-service training or workshop on how to assess these women, we should have more health awareness campaign about such issues like to go to churches, to chief kraals when they are having their gathering, like to the community.’ (Participant 8, female, 26 years old, 3 years’ experience, nursing degree, CHC)

In line with these findings, Sundborg et al. (2012:15–18) mentioned the value of the preparedness of professional nurses to provide quality patient care to women experiencing gender-based violence. In his lecture, Baart (2018) emphasised that spending time with a patient in need helps patients to be free, comfortable and open up. Indeed, gaining trust from the patient requires quality time and a good nurse-patient relationship (Mohammadipour et al. 2017:19).

## Conclusion and implications for practice

It was evident from the findings that the context played a major role in the experience of the participants. They lived in a rural area where gender-based violence is highly prevalent, and where they were exposed to gender-based violence. It was furthermore evident that the concept of caring presence was interwoven in the experience of the participants, for example, that they experienced compassion for the women and they were willing to provide holistic care. However, they experienced reluctance, related to their work environment, their perception of their own competence, their own experience of gender-based violence and their awareness of the beliefs and context of the women. This left them feeling frustrated and with a deepened sense of empathy, as they realised how deeply the women are suffering, whilst at the same time feeling unable to help. They had a need to be guided in providing relational care, as well as a need for strengthened collaboration between multidisciplinary team members.

Recommendations for nursing practice are that attention should be given to the infrastructure of the hospitals, clinics and CHCs to ensure that there are private and safe spaces for women to disclose gender-based violence. To address nurses’ personal experience of gender-based violence and their feelings of being unable to help, debriefing sessions should be provided, reflecting on their own wellness and their experience of connecting with and attuning to the needs of women and providing relational care. To attend to the need for an improved referral system and stronger collaboration between multi-professional team members, regular meetings should be held and action plans should be drawn.

Policy recommendations are that policy should be developed regarding a referral system as well as on guidelines on the assessment and management of women experiencing gender-based violence as relevant in the specific context. Policies should also be developed regarding the wellness of professional nurses providing nursing care to women experiencing gender-based violence.

Recommendations for nursing education are that training should be provided in relational care, caring presence, self-awareness and self-care, a balanced view on theoretical knowledge and inner wisdom and supportive interviewing. Short learning programmes should be developed to tangibly address these topics, and existing modules relating to the ethos of nursing, communication skills and personal growth should specifically give attention to these topics. There should be continuous education on early identification, assessment and management of women experiencing gender-based violence.

Further research is needed to explore the needs of women experiencing gender-based violence in terms of relational nursing care and caring presence. There is a need to explore the views of other nursing categories, multidisciplinary team members and stakeholders on providing holistic and relational care to women experiencing gender-based violence. Further research is needed to explore the views of the husband and extended family on beliefs and culture concerning gender-based violence.
